# Information and Statistical Measures in Classical vs. Quantum Condensed-Matter and Related Systems

**DOI:** 10.3390/e22060645

**Published:** 2020-06-10

**Authors:** Adam Gadomski, Sylwia Zielińska-Raczyńska

**Affiliations:** 1 Institute of Mathematics and Physics (Group of Modeling of Physicochemical Processes), Faculty of Chemical Technology and Engineering, UTP University of Science and Technology, 85-796 Bydgoszcz, Poland; 2 Institute of Mathematics and Physics (Group of Quantum Optics and Information), Faculty of Chemical Technology and Engineering, UTP University of Science and Technology, 85-796 Bydgoszcz, Poland; sziel@utp.edu.pl

**Keywords:** information, entropy, classical vs. quantum system, condensed matter, soft matter, complex systems

## Abstract

The presented editorial summarizes in brief the efforts of ten (10) papers collected by the Special Issue (SI) “Condensed-Matter-Principia Based Information & Statistical Measures: From Classical to Quantum”. The SI called for papers dealing with condensed-matter systems, or their interdisciplinary analogs, for which well-defined classical statistical vs. quantum information measures can be inferred while based on the entropy concept. The SI has mainly been rested upon objectives addressed by an international colloquium held in October 2019, at the University of Science and Technology (UTP) Bydgoszcz, Poland (see http://zmpf.imif.utp.edu.pl/rci-jcs/rci-jcs-4/), with an emphasis placed on the achievements of Professor Gerard Czajkowski (PGC). PGC commenced his research activity with diffusion-reaction (open) systems under the supervision of Roman S. Ingarden (Toruń), a father of Polish synergetics, and original thermodynamic approaches to self-organization. The active cooperation of PGC mainly with German physicists (Friedrich Schloegl, Aachen; Werner Ebeling, Berlin) ought to be underlined. Then, the development of Czajkowski’s research is worth underscoring, moving from statistical thermodynamics to solid state theory, pursued in terms of nonlinear solid-state optics (Franco Bassani, Pisa), and culminating very recently with large quasiparticles, termed Rydberg excitons, and their coherent interactions with light.

The colloquium “What is INFORMATION—How to get, process, code and read it, in the elegant world of mathematical, statistical and quantum physics” which took place on 24–25 October 2019 at the University of Science and Technology (UTP) in Bydgoszcz, Poland, was devoted in part to recognizing the outstanding contribution to statistical thermodynamics and condensed matter physics by Professor Gerard Czajkowski, former director of the Institute of Mathematics and Physics and vice rector for research. Gerard Czajkowski obtained a master’s degree in 1967, Ph.D. in 1971, and habilitation in theoretical physics in 1976, all from Nicolaus Copernicus University in Toruń, Poland. He became a full professor of physics in 1987. All of his thesis topics of interest were devoted to the statistical aspects of thermodynamics, paving the way for quantum information. During fifty years of his academic career, his research interests have evolved from information-theoretical methods in the stochastic theory of open systems to the quantum optics of semiconductors, unlocking a plethora of dynamical effects which might be useful for the practical realization of quantum information processing in solids.

Recent research developments in the fields related to quantum information have become some of the most intriguing investigations in contemporary physics. Many novel concepts nowadays are discussed in the literature, and a broad range of new models of quantum optics and solid-state physics have recently been considered in the context of quantum information theory. A lot of effort has been devoted to investigating new ideas connected to various aspects of quantum correlations, such as entanglement and quantum steering, but also to more practical proposals of the systems, which could be applied in quantum technology. Here, let us juxtapose the main message(s) from ten papers published in the underlying Special Issue (SI).

The paper “Binary Communication with Gazeau–Klauder Coherent States” [1] investigates the advantages and disadvantages of using Gazeau–Klauder coherent states for optical communication. It is proved that using an alphabet consisting of coherent Gazeau–Klauder states related to a Kerr-type nonlinear oscillator instead of standard Perelomov coherent states results in a lowering of the Helstrom bound for error probability in binary communication. The authors also discuss the trace distance between Gazeau–Klauder coherent states and a standard coherent state as a quantifier of distinguishability of alphabets.

Photons are excellent carriers of information, but they are essentially noninteracting with each other in the absence of a dispersive medium. In order to overcome this difficulty, photons should be localized at the characteristic length scale in the medium which enables mapping photons into medium excitations, preserving their phase relations. Electromagnetically induced transparency (EIT) is a phenomenon which enables the transference of coherence and quantum states from light to collective medium spin excitations and has turned out to be very attractive for applications in quantum information. Solid bulk media are systems well worth considering for storing and processing quantum information because they have a number of advantages over atomic gases. Recently, Rydberg excitons have attracted a great deal of attention due to their exciting features: the distinct combination of their long radiative lifetimes, sensitivity to external fields, and strong dipolar interactions could be exploited to realize quantum interfaces for quantum information processing.

In the paper entitled “Electromagnetically Induced Transparency in Media with Rydberg Excitons 1: Slow Light” [2], it is shown that electromagnetically induced transparency (EIT) can be realized in media with Rydberg excitons. With realistic, reliable parameters which show good agreement with optical and electro-optical experiments, as well as the proper choice of Rydberg exciton states in cuprous oxide crystal, the author indicates how EIT can be achieved. The calculations show that, due to a large group index, one can expect the slowing down of a light pulse by a factor of about 10^4^ in this medium.

The paper “Electromagnetically Induced Transparency in Media with Rydberg Excitons 2: Cross-Kerr Modulation” [3] discusses the issue of nonlinear interaction of photons in the linear regime of EIT. By mapping photons into the sample of cuprous oxide with Rydberg excitons, it is possible to obtain a significant optical phase shift due to third-order cross-Kerr nonlinearities realized in a transparent medium. The optimum conditions for observation of the phase shift over π in Rydberg excitons media are examined. A discussion of the application of the cross-phase modulations in the field of all-optical quantum information processing in solid-state systems is presented.

The proposal of matching fractal plasmons, a quantum or quasiparticle associated with a local collective oscillation of charge density, with information processing is discussed in “Fractal Plasmons on Cantor Set Thin Film” [4]. The propagation of surface plasmon–polaritons is investigated in a metallic, fractal-like structure based on the Cantor set. This work uncovers a novel opportunity to elicit information about the surface structure from the reflection spectrum of surface plasmon–polaritons and thereby establishes a link between optical properties and different fractal geometries.

Another paper referring to plasmonics, “Interaction and Entanglement of a Pair of Quantum Emitters near a Nanoparticle: Analysis beyond Electric-Dipole Approximation” [5], considers the problem of interactions between quantum particles positioned near plasmonic nanostructures which may be substantially stronger than in free space, leading to fast energy exchange between the particles and corresponding fast entanglement generation. The authors study the influence on these quantities of interaction channels beyond the electric dipole. It may not only be significant in terms of numbers, but may also affect the spatial symmetry of the optical response as a function of the position of particles with respect to the plasmonic nanostructure.

The first of the classical statistical papers [6] of the underlying SI is about the profound misuses, misunderstanding, misinterpretations, and misapplications of entropy, the Second Law of Thermodynamics and Information Theory. It points, in the author’s opinion [6], to the “Greatest Blunder Ever in the History of Science”. It is not about a single blunder admitted by a single person (e.g., Albert Einstein’s assertions on the cosmological constant), but more a blunder of immense proportions, the claws of which have spread over all branches of science; from thermodynamics, cosmology, and biology to sociology and much more.

The second from the series of classical statistical papers [7] embarks on a versatile analysis of the conformation of albumin in the temperature range of 300–312 K, i.e., in the physiological range. By employing molecular dynamics simulations, values of the backbone and dihedral angles for this molecule are calculated. An analysis is performed on the global dynamic properties of albumin treated as a chain. In this temperature range, the parameters of the molecule and the conformational entropy derived from two angles that reflect global dynamics in the conformational space are obtained. A rationale based on scaling theory for the subdiffusion Flory–De Gennes type exponent of 0.4 is unfolded in conjunction with detecting the most appreciable fluctuations of the corresponding statistical test parameter. These fluctuations are shown to coincide adequately with entropy fluctuations, namely, the oscillations around the thermodynamic equilibrium. By applying the Kullback–Leibler theory, differences between the distribution of the root-mean-square displacement for each temperature and time window are addressed.

The third from the series of classical statistical papers [8] regards the problem of information processing toward living organisms while based on chemical reactions. It is worth noting that the human achievements in constructing chemical information processing devices demonstrate that it is difficult to design such devices using the bottom-up approach. Therefore, an alternative top-down design of a network of chemical oscillators that performs a selected computing task is worth pursuing. As an example, a simple network of interacting chemical oscillators that operates as a comparator of two real numbers is analyzed. The information on which of the two numbers is larger is coded in the number of excitations detected on oscillators forming the network. The parameters of the network are optimized to perform this function with maximum accuracy. A discussion is carried out on how information theory methods can be applied to obtain the optimum computing structure.

The next from the series of classical statistical papers [9] studies the flow of a substance in a channel network which consists of nodes of the network and edges which connect these nodes and form paths for motion of the substance. The channel can have an arbitrary number of arms, and each arm can contain an arbitrary number of nodes. The flow of the substance is modeled by a system of ordinary differential equations. First, a model for a channel with arms each involving an infinite number of nodes is discussed. For the stationary regime of motion of a substance in such a channel, the probability distributions connected to the distribution of the substance in any of channel’s arms and in the entire channel are derived. The obtained distributions can be connected to the Waring distribution. Next, a model for the flow of a substance in a channel with arms each containing a finite number of nodes is analyzed. The probability distributions connected to the distribution of the substance in the nodes of the channel for the stationary regime of flow of the substance are obtained. These distributions are new, and based on them the corresponding information measure and Shannon information measure for the studied kind of flow of a substance are derived.

The last work from the series of classical statistical papers [10] collected by the SI addresses in a biomimetic way the subject of drones and drone swarms, equipped with sophisticated algorithms that help them achieve mission objectives. Such algorithms vary in their quality such that their comparison needs a metric that would allow for their correct assessment. The novelty of this study relies on analyzing, defining, and applying to swarms the construct of cross-entropy, known from thermodynamics and information theory. It can be used as a synthetic measure of the robustness of algorithms that can control swarms in the case of unexpected obstacles and unforeseen problems. This work applies in terms of necessary formalizations (algorithms) and, by addressing a few examples, to collision avoidance issues when prompted to react to material obstacles.

To sum up, this SI accumulates substantial and versatile evidence on the widely distributed phenomenon of information transmission and how to measure it from a number of fairly complex entropic systems, ranging from classical to quantum (albeit discussed purposely in a reversed order), that have been thoroughly studied by the contributors of the presented SI [1,2,3,4,5,6,7,8,9,10]. The studies by PGC and coworkers have, to a great extent, enabled the extraction of many useful information measures from the systems covered by the SI.
